# Exaggerated Intergroup Bias in Economical Decision Making Games: Differential Effects of Primary and Secondary Psychopathic Traits

**DOI:** 10.1371/journal.pone.0069565

**Published:** 2013-08-08

**Authors:** Steven M. Gillespie, Ian J. Mitchell, Ian Johnson, Ellen Dawson, Anthony R. Beech

**Affiliations:** 1 Centre for Forensic and Criminological Psychology, School of Psychology, University of Birmingham, Birmingham, United Kingdom; 2 School of Psychology, University of Birmingham, Birmingham, United Kingdom; Hungarian Academy of Sciences, Hungary

## Abstract

Psychopathic personality traits are linked with selfish and non-cooperative responses during economical decision making games. However, the possibility that these responses may vary when responding to members of the in-group and the out-group has not yet been explored. We aimed to examine the effects of primary (selfish, uncaring) and secondary (impulsive, irresponsible) psychopathic personality traits on the responses of non-offending participants to the in-group and the out-group (defined in terms of affiliation to a UK University) across a series of economical decision making games. We asked a total of 60 participants to act as the proposer in both the dictator game and the ultimatum game. We found that across both tasks, those who scored highly for secondary psychopathic traits showed an elevated intergroup bias, making more generous offers toward members of the in-group relative to the out-group. An exaggerated intergroup bias may therefore represent a motivational factor for the antisocial behavior of those with elevated secondary psychopathic traits.

## Introduction

The term psychopathy refers to a severe disorder of personality, characterized by callousness and a lack of care for others, poor empathic functioning and a lack of remorse or guilt [1,2]. The presence of such interpersonal and affective abnormalities differentiates psychopathy from other syndromes characterized by marked levels of criminality and aggression, including ‘sociopathy’ and antisocial personality disorder (ASPD) [3,4]. For example, ASPD, as defined in the *Diagnostic and Statistical Manual of Mental Disorders* [5], refers to a set of behavioral criteria including aggression toward people or animals, destruction of property, deceptiveness or stealing and serious rule violations. However, with the exception of one item (absence of remorse), these criteria do not reference the hallmark interpersonal/affective features of psychopathy. Furthermore, it has been debated whether or not criminally antisocial behavior is central to the syndrome of psychopathy or merely a downstream correlate of the underlying personality features [6–8].

In support of a theoretical distinction between psychopathy and ASPD, findings indicate that offenders with ASPD plus psychopathy show a more severe pattern of offending relative to those with ASPD in the absence of psychopathy, and those with neither diagnosis [9]. Additional evidence points to differences in the processing of emotional stimuli between psychopaths and non-psychopaths with ASPD [9,10]. For example, psychopathy deficits in event related brain potentials during a Go/No-Go task have been revealed, indicating blunted processing of emotionally negative words among psychopaths relative to non-psychopaths with ASPD [10]. However, variants of clinically diagnosable psychopathy have also been suggested, with the most common distinction made between ‘primary’ and ‘secondary’ subtypes [11].

Primary and secondary subtypes of psychopathy may be differentiated on the basis of levels of neuroticism and anxiety, with the secondary variant failing to resemble Cleckley’s traditional description in some important respects [11,12]. While primary psychopaths present with low levels of trait anxiety the opposite is true for secondary psychopathic individuals [13]. In support of this distinction, differences in electrodermal skin responses have been noted during aversive conditioning trials with primary and secondary psychopathic individuals [14]. Furthermore, cluster analytical methods with samples of offenders and non-offenders also provide evidence for a primary/secondary distinction in psychopathic personality [15,16].

In her seminal article on the ‘sociobiology of sociopathy’, Mealey [17,18] outlines a game theoretic model for anti-sociality. In particular, Melaey [17,18] refers to two subtypes of sociopath: ‘primary’ and ‘secondary’. It should be noted that while the primary subtype most closely resembles traditional descriptions of psychopathy [1,2] the secondary variant may more closely conform to the criteria for ASPD, or descriptions of secondary psychopathy. As such, although both primary and secondary subtypes present with high levels of antisocial behavior, it is suggested by Mealey [17] and others [11,12], that the behavior of these subtypes may be differentially motivated. Consistent with traditional descriptions of psychopathy [1,2], Mealey argues that primary psychopaths antisocial behavior stems from high levels of callous unemotionality and a lack of remorse for others [17,18]. In addition, it is argued that these traits may reflect a genetic predisposition toward psychopathy [17], a position which has received recent support [19,20]. In contrast, it is suggested that the antisocial deviance of secondary sociopaths may stem from adverse early experiences, including an abusive and neglectful childhood environment. This early background may leave the individual at an evolutionarily competitive disadvantage. Thus, selfish behaviors are selected which allow the individual to compete for resources.

The use of such selfish and non-cooperative behaviors may be tested under controlled circumstances using game theoretic tasks. Such tasks include the Prisoner’s Dilemma Game (PDG), which has been used to test the use of cooperative and non-cooperative strategies in relation to psychopathic personality. For example, it has been shown that psychopaths have a strong tendency to make competitive, non-cooperative responses compared with non-offenders [21]. Furthermore, these non-cooperative responses were also found to yield higher individual rewards. The PDG has also been used to test the use of non-cooperative strategies among adults with psychopathic tendencies [22,23].

Mealey [17,18] suggests that primary psychopaths, lacking in interpersonal emotions including empathy, guilt and loyalty, may adopt a social interactional style characterized by a fixed antisocial strategy. Although the use of a fixed antisocial strategy may be at odds with descriptions of primary psychopaths as manipulative and conning, it is suggested that the primary psychopath uses a cost-benefit approach to achieve immediate personal gain. As such, the use of conning and deceitful strategies may be of greatest benefit under circumstances where an immediate pay-off for antisocial strategies is unlikely, leading to the use of deceitful pro-social strategies. However, as highlighted by Mealey [17,18], the fixed use of cheating strategies may have long term losses under circumstances where continued social interactions occur. For example, where a player develops a reputation for defection interactions may become less frequent, thereby limiting the opportunity for future profit. Thus, while the fixed use of one antisocial strategy may be characteristic of the primary psychopath, the secondary psychopath may display cooperative and non-cooperative strategies dependent upon environmental circumstances. One such evolutionarily important environmental circumstance may be the in-/out-group status of the person with whom a social interaction occurs.

From an evolutionary perspective, the presence of a strong and faithful in-group may be of particular importance for those who are at a competitive disadvantage. As such, the secondary psychopath could show a heightened intergroup bias in the way that they allocate resources to the in-group and the out-group. These interactions may be characterized either by in-group liking or out-group derogation, both of which either directly or indirectly serve to promote the needs of the in-group and aid survival of its members [24,25]. Acts of in-group liking, for example allocating generous amounts of resources to the in-group, would serve to strengthen one’s own group. Furthermore, a selfish allocation of resources to the out-group would serve to undermine the power of the out-group, protecting the needs of and strengthening the position of the in-group [25]. Secondary psychopaths cheating behaviors may therefore be characterized by parochial altruism, with parochial acts of aggression and selfishness directed toward the out-group, and altruistic acts of generosity and pro-sociality characterizing in-group social interactions.

Although such selfish strategies may be most prevalent among those who present with high levels of antisocial deviance, they may nonetheless be detected sub-clinically under immediate environmental circumstances where pro-social strategies are less profitable. While psychopathy in a forensic context is most commonly assessed using the Psychopathy Checklist – Revised (PCL-R) [2,26], psychopathic personality traits may nonetheless be observed in sub-clinical populations through the use of self-report psychopathy scales. One such scale, the Levenson Self Report Psychopathy Scale (LSRP) [27], was designed to parallel the two factor structure of the PCL-R. Thus, while the primary subscale of the LSRP includes items relating to selfishness and a lack of care for others, the secondary subscale includes items which tap a proclivity to boredom, recklessness, and a lack of responsibility for one’s own actions. The factor structure and construct validity of the LSRP has been examined in a sample of 549 male inmates from Wisconsin state prisons [28]. Results showed modest support for the original two factor structure and medium sized correlations of the primary and secondary subscales with the corresponding factors of the PCL-R [28]. It has been suggested however that criteria which load on to Factor 2 of the PCL-R are highly overlapping with symptoms of ASPD. For example, significant correlations have been noted of total ASPD symptoms and of ASPD diagnoses with PCL-R Factor 2 scores in a sample of 313 male inmates of the Federal Correctional Institution in Tallahassee, Florida [29]. As such, the secondary subscale of the LSRP may tap psychopathic personality traits which are closely related to those of ASPD.

Similar to those with a diagnosis of ASPD, psychopathic individuals and those with psychopathic tendencies are generally assumed to behave in an antisocial and selfish manner. As well as the PDG, social cooperation has been investigated in psychopaths using experimental games including the dictator game and the ultimatum game. These laboratory based games typically involve participants deciding how to carve up imaginary rewards between themselves and a competitor. The distribution of rewards is governed by different reinforcement contingencies which enable participants to display varying levels of generosity and altruism, as well as selfishness and spite [30].

In the dictator game, a player determines how a reward should be split between themselves and a second, passive player. In contrast, players in the ultimatum game must take on the role of either the proposer or the responder. The proposer is required to split a monetary amount between themselves and the responder, who may either accept or reject this offer. If the responder accepts this single offer, the money is split as proposed. If the responder rejects the offer, neither player receives anything.

The dictator game and ultimatum game have been used to probe social cooperativeness in primary and secondary convicted psychopaths [31]. Primary psychopaths showed significantly lower acceptance rates of unfair ultimatum offers and proposed lower amounts in the dictator game. A similar pattern of responding was seen in a separate group of participants composed of patients with lesions of the ventro-medial prefrontal cortex, but not in secondary psychopaths [31]. These results reinforce the view that those with secondary psychopathy do not show a fixed pattern of non-cooperative responding, but rather may employ differing strategies dependent on the requirements of the social interaction at hand.

In the current study we worked with non-offenders to explore the effects of primary and secondary psychopathic traits on social cooperativeness when interacting with in-groups and out-groups in the dictator game and the ultimatum game. We hypothesized that high primary psychopathic traits would be associated with selfishness and reduced cooperation, independent of the in-/out-group status of the other player. In contrast, we hypothesized that those with high secondary psychopathic traits would show higher levels of generosity during interactions with other in-group members, while out-group interactions would be characterized by selfish non-cooperation.

## Materials and Methods

### Ethics statement

The current research was approved by the University of Birmingham Committee for Ethical Review. All participants were asked to sign their written informed consent prior to participation.

### Participants

A total of 60 participants (50 female), with a mean age of 19 (range = 18-23, SD = 1.0) were recruited from the University of Birmingham, UK. Participants received course credit in return for their participation.

### Measures

The Levenson Self-Report Psychopathy Scale (LSRP) [27] was completed by all participants as part of a battery of questionnaire based measures. The LSRP, designed for the measurement of psychopathic personality traits in non-institutionalized populations, consists of a 16 item primary subscale and a 10 item secondary subscale. While the primary subscale taps the selfish and uncaring characteristics associated with Factor 1 of the PCL-R, the secondary subscale measures behavioral and lifestyle factors associated with Factor 2, including boredom and impulsivity. Adequate internal validity of the LSRP has been demonstrated in a sample of 487 undergraduate psychology students, with a Cronbach’s alpha of .82 for the primary subscale and .63 for the secondary subscale, which was considered adequate for a ten item scale [27].

### Procedure

We used two separate tasks to assess the intergroup bias in relation to primary and secondary psychopathic traits: a computerized dictator game, a computerized ultimatum game. All tasks were prepared using E-Prime 2.0 stimulus presentation software. Participants always completed the dictator game prior to the ultimatum game. This order was enforced as proposed amounts during dictator game trials are typically dictated by generosity. However, ultimatum game trials require more careful thought as offers may either be accepted or rejected. Thus, we wished to avoid a scenario in which participants continued to make carefully judged proposals on the dictator game as a result of having first completed the ultimatum game.

#### The Dictator Game and Ultimatum Game

Prior to the first dictator game trial, participants received on-screen instructions that they were to split an amount of £10 between themselves and an on-screen player. The minimum amount a participant could allocate was £1, with a maximum allocation of £9. In order to maximize personal gains, participants may be expected to propose only a minimal amount to the on-screen player. However, higher proposals may represent attempts to appear generous. Following completion of all dictator game trials, participants were presented with additional instructions for ultimatum game proposals. Participants were again instructed to split an amount of £10 between themselves and the on-screen player. However, participants were informed that their offers could subsequently be accepted or rejected by the respective on-screen players. If an offer was rejected, participants were instructed that both they and the on-screen player would receive nothing.

**Figure 1:**
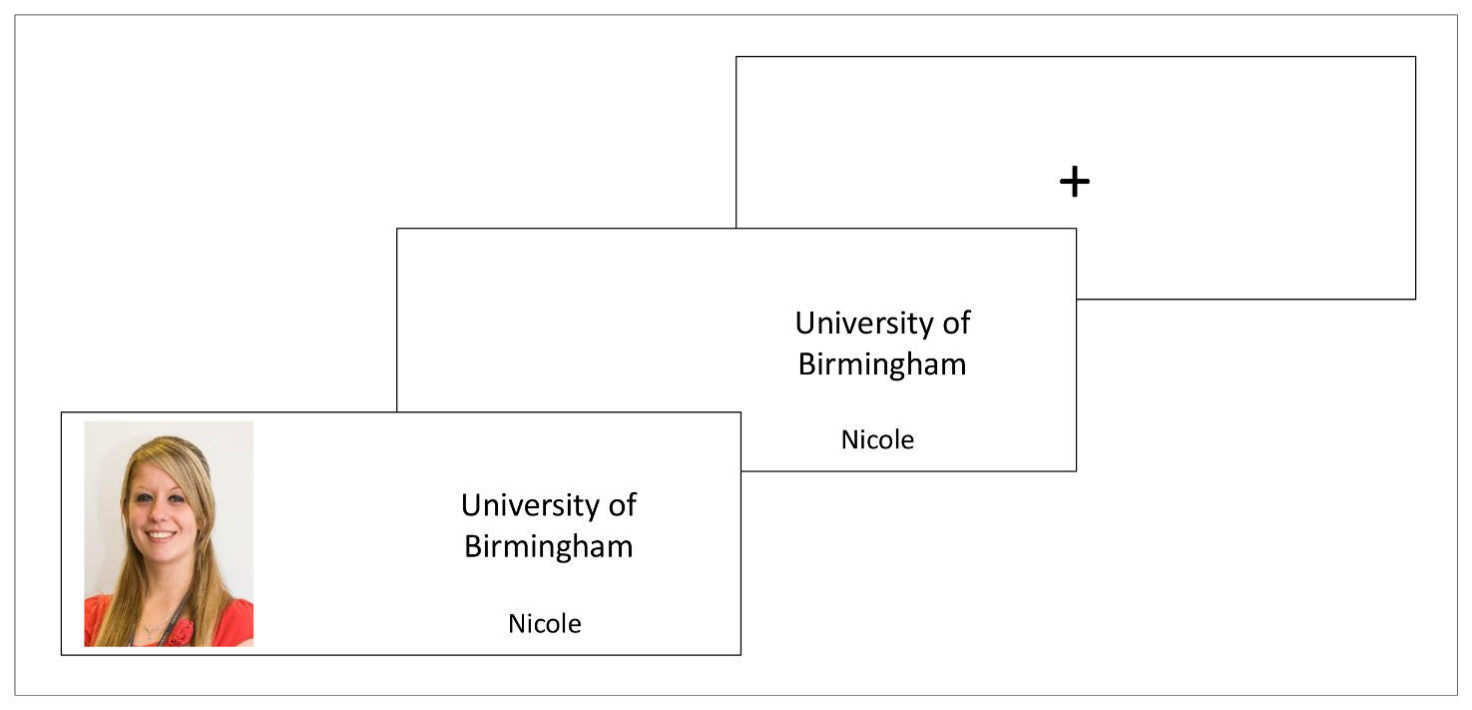
Overview of the dictator game and the ultimatum game. For each of 38 trials participants view a central fixation cross for 500ms. This is followed by a 500ms presentation of a false forename and University logo which primes the same (University of Birmingham) or other (University of Manchester) University affiliation. University logos are then joined by a picture of the person to whom participants believe they are to make an offer. Once participants make an offer the next trial begins. Note: experimental trials included corporately formatted logos and text, not displayed above.

A total of 38 Caucasian faces (10 female) were selected from the PUT face database [32] for use in the dictator game and ultimatum game. Each face was paired with a forename and allocated to either the in-group or the out-group using a same or other university manipulation. This was achieved through presentation of either same (University of Birmingham) or other (University of Manchester) university logos, including corporately formatted names and crests, alongside each face and forename (see Figure 1; please note that the photograph used in the figure is not the original image used in the study, but a similar image used for illustrative purposes only. The subject of the photograph has given written informed consent, as outlined in the PLOS consent form, to publication of their photograph).

Participants were informed that the current research was a collaborative investigation with the University of Manchester, assessing the impact of differing personality factors on economical decision making. Both the information sheet and consent forms included the name and official crests of both the University of Birmingham and the University of Manchester. Each task consisted of 38 trials. For each trial, participants were presented with a central fixation cross for 500ms. The fixation period was followed by a 500ms presentation of a university logo indicating same (University of Birmingham) or other (University of Manchester) university affiliation, alongside a false forename. University affiliation primes were subsequently joined by the image of a face to whom participants were to make a proposed split while acting as the proposer in the dictator game and the ultimatum game. The trial was terminated when participants indicated the proposed amount to be allocated to the on-screen player.

## Results

### Psychopathy

The LSRP was used to measure psychopathic personality traits in the current sample. Participants demonstrated a mean score on the primary subscale of 28.6 (SD = 6.39), ranging from 16 to 44. The mean score for the secondary subscale was 20.2 (SD = 4.48), with a range of 12 to 36. We noted that the mean primary and secondary psychopathy scores for the present study fall toward the lower end of the range of mean scores reported in previous studies using the LSRP with non-offending samples [27,33,34]. Furthermore, we compared LSRP scores for the current sample of non-offenders with those obtained from a sample of 549 male inmates from Wisconsin state prisons (28), for primary psychopathy, M = 32.99 (SD = 8.19), and secondary psychopathy, M = 21.68 (SD = 5.05). These comparisons revealed that scores recorded on both subscales were higher among the offending sample, with a medium Cohen’s *d* effect size of 0.55 for the primary subscale, and a small effect size of 0.3 for the secondary subscale. In contrast to previous findings with the LSRP, the primary and secondary sub-scales were not found to be significantly positively correlated (*r* = .18, *p* > .05). The primary subscale yielded good internal reliability, with a Cronbach’s alpha estimate of .86. The secondary subscale also showed adequate internal reliability, with a Cronbach’s alpha estimate of .71.

### Dictator game proposals

Dictator game proposals toward the in- and the out-group were calculated for all participants. A paired samples t-test proved to be non-significant (*t* = 1.59, *p* > .05) with no differences in responses for the in-group, M = £3.95 (out of a possible maximum of £9) (SD = 1.43) compared to the out-group, M = £3.77 (SD = 1.34), Cohen’s *d* effect size = 0.13. A Pearson correlation co-efficient showed that participants responses to the in- and out-group were positively correlated (*r* = .81, *p* < .001).

### Ultimatum game offers

Average offers to the in- and out-group were calculated for each participant. A paired samples t-test indicated a significant effect of in-/out-group (*t* = 3.17, *p* < .01) with participants offering on average a fairer split for the in-group, M = £4.73 (SD = .82), compared to the out-group, M = £4.55 (SD = .86). A Cohen’s *d* effect size calculation of 0.22 suggests a small effect of in-/out-group status on ultimatum offers. Pearson’s correlation co-efficient showed that participants responses to the in-group and the out-group were positively correlated (*r* = .87, *p* < .001).

### Method for analysis

To investigate the effects of level of primary psychopathic traits on dictator and ultimatum game proposals a mean split was used to divide participants in to a low scoring group (*n* = 28; mean primary psychopathy score = 23) and a high scoring group (*n* = 32; mean primary psychopathy score = 34). We used two separate mixed model ANCOVAs with the factors affiliation (in-group, out-group) and level of primary psychopathy (low, high) with secondary psychopathy as a covariate. Analyses were repeated for secondary psychopathic traits, with participants divided about the mean in to low (*n* = 38; mean secondary psychopathy score = 18) and high (*n* = 22; mean secondary psychopathy score = 25) scoring groups, with primary psychopathy included as a covariate. Effect sizes are reported as partial-eta squared with the following suggested norms for interpretation: small = .01; medium = .06; large = .14.

### Effects of primary psychopathy

#### Dictator game proposals

**Figure 2:**
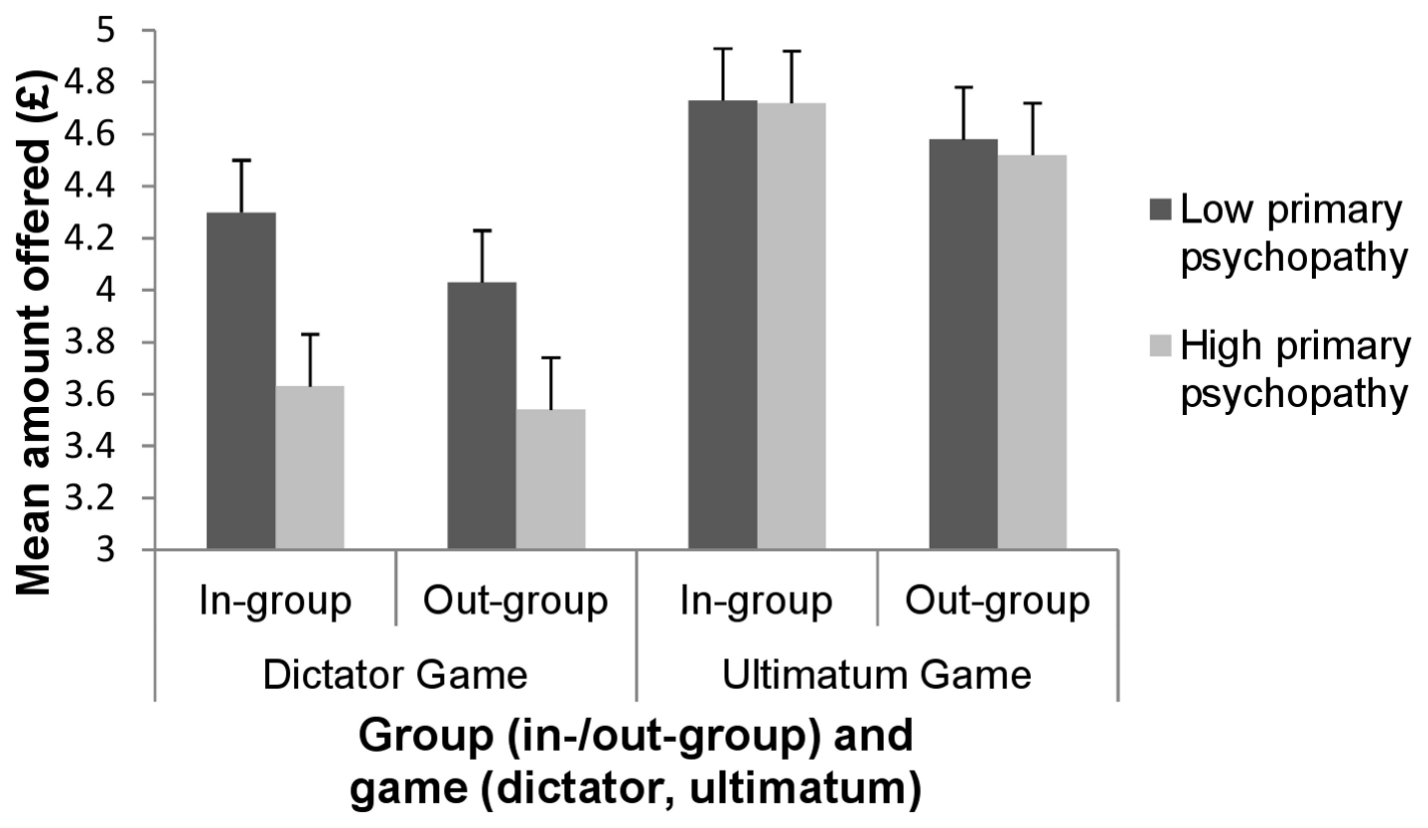
Responses on dictator and ultimatum game for low and high primary psychopathy groups. We observed no differences in the average amounts offered to members of the in-group and the out-group for participants in the low and the high scoring primary psychopathy groups, in either the dictator game or the ultimatum game.

We showed that there were no differences in giving behavior to members of the in- and the out-group *F*(1,57) = .21, *p* > .05, pη² = .004. We also failed to find an effect of level of primary psychopathic traits on giving behavior *F*(1,57) = 2.54, *p* > .05, pη² = .04 or interaction of level of primary psychopathic traits with giving behavior to the in- and the out-group *F*(1,57) = .84, *p* > .05, pη² = .02 (see Figure 2).

#### Ultimatum game offers

In contrast to dictator proposals, ultimatum game offers may be accepted or rejected, with rejection resulting in both parties receiving no money. Similar to results for dictator game trials, we observed no significant effects of in-/out-group status *F*(1,57) = .15, *p* > .05, pη² = .003 nor main effect of level of primary psychopathic traits *F*(1,57) = .93, *p* > .05, pη² = .00. We found no differences in giving behavior to the in- and the out-group for low and high scoring primary psychopathic traits participants *F*(1,57) = .08, *p* > .05, pη² = .001 (see Figure 2).

### Effects of secondary psychopathy

#### Dictator game proposals

**Figure 3:**
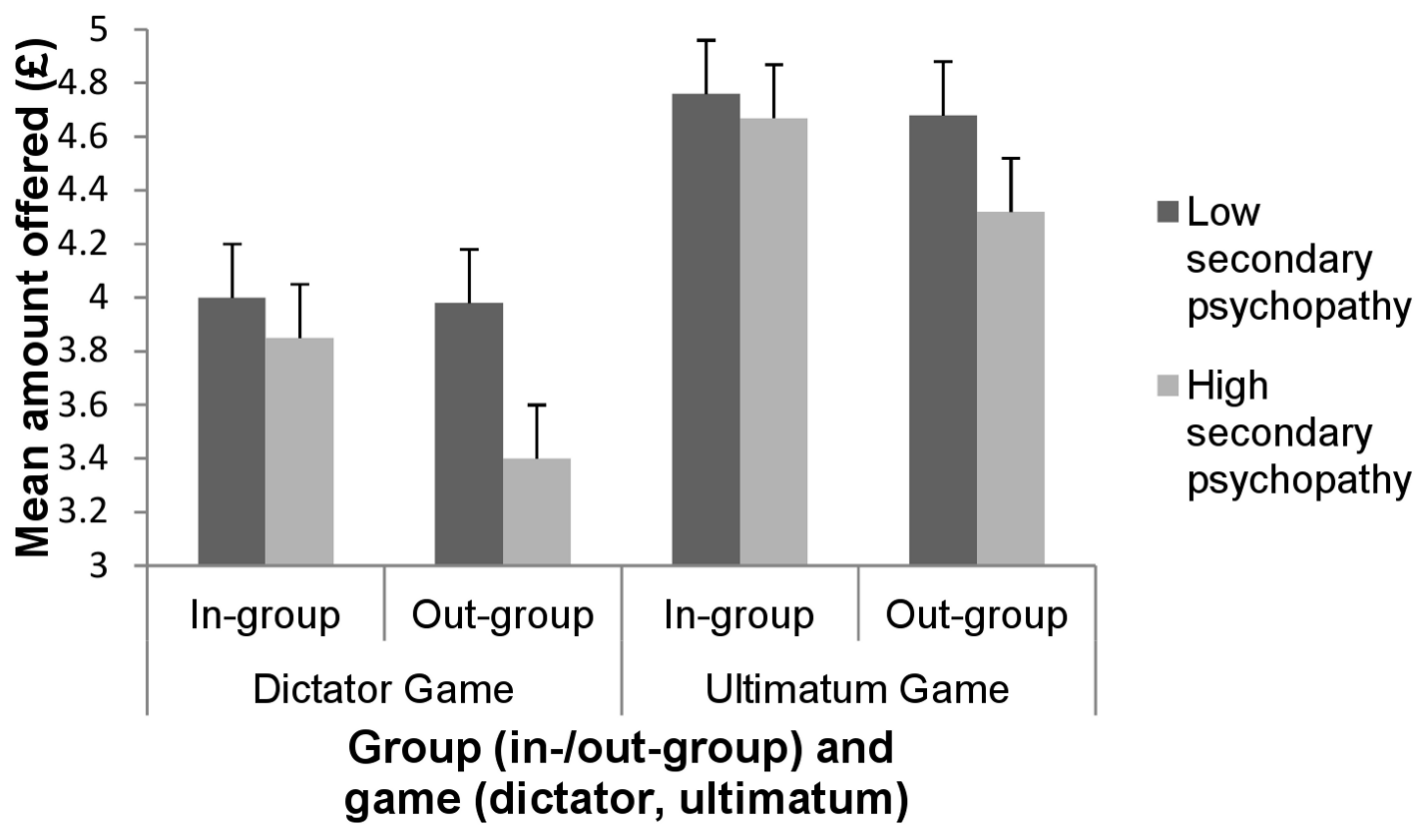
Responses on dictator and ultimatum game for low and high secondary psychopathy groups. We found that in both the dictator game and the ultimatum game, participants in the high scoring secondary psychopathy group offered smaller amounts to members of the out-group relative to the in-group. However, participants in the low scoring secondary psychopathy group offered similar amounts to both members of the in-group and the out-group across both the dictator game and the ultimatum game.

We showed that there were no differences in giving behavior to members of the in- and the out-group *F*(1,57) = 2.24, *p* > .05, pη² = .04, or between those with low and high levels of secondary psychopathic traits *F*(1,57) = .84, *p* > .05, pη² = .02. We did however show an interaction of in-/out-group with level of secondary psychopathic traits *F*(1,57) = 4.19, *p* < .05, pη² = .07, whereby those in the high scoring group proposed lower amounts for members of the out-group relative to the in-group (see Figure 3). However, both low and high scoring secondary psychopathic traits participants made similarly fair offers for members of the in-group. These results therefore suggest a pattern of out-group derogation, rather than in-group liking, among those with high secondary psychopathic traits.

#### Ultimatum game offers

We showed that there were no differences in giving behavior to members of the in- and the out-group *F*(1,57) = .001, *p* > .05, pη² = .000 or between low and high scoring secondary psychopathic traits participants *F*(1,57) = 1.12, *p* > .05, pη² = .02. We did however show an interaction of in-/out-group with level of secondary psychopathic traits *F*(1,57) = 5.02, *p* < .05, pη² = .08. Again, both low and high scoring secondary psychopathic traits participants offered similarly fair amounts to members of the in-group. However, participants in the high secondary psychopathy group proposed lower amounts to those in the out-group relative to the in-group, indicating a pattern of out-group derogation (see Figure 3).

## Discussion

In the current study we aimed to investigate the effects of primary and secondary psychopathic personality traits on economical decision making to members of the in- and the out-group. We predicted that primary psychopathic traits, which reflect selfishness and a lack care for others, would be associated with a pattern of selfish responding in the dictator game and the ultimatum game, with both tasks necessitating similar decisions. On the other hand, we predicted that secondary psychopathic traits would be linked with differential response patterns for members of the in- and the out-group. More specifically, we predicted that offers to out-group members would be characterized by a more selfish response pattern.

Our results showed no differences in dictator or ultimatum game offers, irrespective of in-/out-group status, between participants who scored high and low on primary psychopathic traits. However, these findings may reflect low levels of primary psychopathic traits among sub-clinical samples. For example, low levels of psychopathic personality traits have been reported among the general household population of Great Britain [35] and across world regions [36]. Furthermore, rates have been shown to be lower among females relative to males [35,36]. Nonetheless, primary psychopathic trait scores in the current sample were found to be within the range of scores reported elsewhere for non-offenders on the primary subscale of the LSRP [27,33,34] and differed from the scores obtained in an offending male sample [28] by a medium effect size. As such, we would suggest that ours is a representative sample for research on psychopathic personality traits among non-offenders.

Although counter to our prediction, the absence of an effect of primary psychopathic traits on generosity in the ultimatum game is consistent with earlier work showing that convicted primary psychopaths offered similar amounts as healthy controls [31]. However, in contrast to responses in the ultimatum game, these convicted primary psychopaths did demonstrate a pattern of selfish responding on dictator game trials [31]. The results of the current study, as well as those outlined above, are contrary to early hypothesizing on the nature of responding in game theoretic models of psychopathy [17]. Such models suggest that primary psychopathic traits should be linked with a fixed and selfish pattern of responding, which is independent of the specific circumstances of the interaction.

The finding that convicted primary psychopaths show a selfish pattern of responding under dictator game but not ultimatum game conditions [31] may be explained by the need for a fairer pattern of responding in the ultimatum game. While selfish ultimatum game offers are often rejected resulting in both players receiving nothing, selfish responses on the dictator game go unpunished. Thus, a selfish response pattern still allows for a monetary gain during the dictator game, but not the ultimatum game.

In the current experiment we also expected that the self-centeredness associated with primary psychopathic traits would manifest itself independent of the in-/out-group membership of the on-screen player. Our results show that there was no interaction between level of primary psychopathic traits and generosity of proposals to members of the in-group and the out-group. These findings are therefore supportive of the hypothesis that the pattern of responding in those with high primary psychopathic traits would not be dependent up on the specific environmental or situational demands of the interaction. These findings may be consistent with traditional conceptualizations of psychopathy which refer to emotional-interpersonal deficits, including pathologic egocentricity, incapacity for love, and unresponsiveness in general interpersonal relations [1]. As such, those with high primary psychopathic traits may be less likely to form a close in-group to whom they would feel any great sense of loyalty.

In contrast to primary psychopathic traits which refer to affective and interpersonal features, secondary traits tap behavioral instability and social deviance. In accordance with Mealey’s game theoretic model of psychopathy [17,18], it was expected that individuals characterized by secondary psychopathic traits would exhibit increased generosity for the in-group, and/or selfish, non-cooperative behavior when dealing with members of the out-group. Although we showed that there were no differences in giving behavior between high and low scoring participants on either the dictator or the ultimatum game, we did observe significant differences in dictator and ultimatum game proposals to members of the in-group and the out-group, between low and high scoring participants on secondary psychopathic traits.

Dictator game results indicated that participants with the highest levels of secondary psychopathic traits showed decreased generosity toward out-group relative to in-group members. In contrast, low scoring participants showed similar levels of generosity toward both in- and out-group members. This pattern of results is indicative of a link between secondary psychopathic personality traits and out-group derogation. Since proposals in the dictator game can neither be accepted nor rejected, giving behavior is presumed to reflect altruism or guilt. The implication is that those with elevated levels of secondary psychopathic traits may feel less guilt and show lower levels of generosity to members of the out-group. Participants in the high scoring secondary psychopathic traits group also showed reduced generosity for members of the out-group while making ultimatum game offers.

Our results suggest that individuals with elevated levels of secondary psychopathic traits may adapt a strategy whereby reasonable amounts are offered while interacting with members of the in-group. Such a strategy would aid the development of a reputation for cooperation among those with whom future interactions are most likely [17]. Such a reputation may aid the development of a close in-group, which might allow those with high secondary psychopathic traits to compete more effectively for resources in the future [24,25]. In contrast, interactions with members of the out-group are more likely to be infrequent and far between. Thus, a selfish pattern of responding toward members of the out-group may lead to financial gain without reducing the opportunity for future, potentially profitable interactions.

It is possible that these findings in relation to generosity will equally well apply to other interpersonal and social emotions, including empathy, sympathy and guilt, when dealing with members of the in-group and the out-group. Consistent with a callous and unemotional affective style, it may be hypothesized that high primary psychopathic traits would be associated with reduced feelings of empathy, guilt and remorse for members of both the in- and the out-group. Conversely, secondary psychopathic traits may be linked with normal or enhanced levels of such interpersonal emotions for members of the in-group, while presenting with severely reduced levels for members of the out-group.

These findings may relate to recent findings in the neuroscience literature. For example, it has been indicated that the neuropeptide oxytocin may be implicated in the expression of interpersonal emotions including trust [37], generosity [38] and envy and gloating [39], as well as intergroup conflict [40] and in-group liking [41]. Consistent with a link between secondary psychopathy and reduced generosity to the out-group, it has been found that oxytocin levels are severely elevated among psychopathic patients [42]. More specifically, a positive correlation of levels of oxytocin with Factor 2 scores on the PCL-R has been noted [42]. Thus, an elevated intergroup bias among those with high Factor 2 scores may be associated with heightened levels of oxytocin.

Although it remains unclear how the results of the current study may relate to clinically relevant forms of psychopathy, it has been noted that psychopathic personality most likely refers to a continuum and not a discrete category of individuals [43–45]. Thus, the dimensional nature of the psychopathy construct might suggest that the results of the present study may be particularly exaggerated for those at the extreme high end of the psychopathy continuum. As such it may be hypothesized that the intergroup bias may be particularly elevated among those scoring highly for the lifestyle and antisocial features of psychopathy, with such traits paralleled in the secondary subscale of the LSRP. However, we would urge caution in making extrapolations to clinically relevant forms of psychopathy on the basis of psychopathic personality trait information.

It has also been highlighted that the use of self-report scales for the measurement of psychopathic traits may be problematic given the high levels of dishonesty, malingering, and deceitfulness inherent in psychopathic personality [46]. However, evidence suggests that psychopaths show lower levels of socially desirable responding and positive impression management tendencies [47,48]. Furthermore, psychopathic personality has been shown to be unrelated to malingering success [49,50]. As such, although caution may be necessary when interpreting results based on self-reported psychopathy, evidence suggests that such tools are a valid means of assessing psychopathic personality.

To summarize, while secondary psychopathic traits were consistently associated with elevated levels of antisocial sentiment for the out-group relative to the in-group, this pattern was largely absent in association with primary psychopathic traits across the two tasks. These results are in-keeping with the hypothesized distinction between members of the in- and out-group in relation to secondary psychopathic traits. The results of the current investigation also suggest a need to investigate the role of secondary psychopathic traits in relation to offending behaviors which are driven by loyalty to an in-group and/or hatred of the out-group. Such crimes would include those committed as part of a gang or other social group and so called ‘race hate’ crimes.
